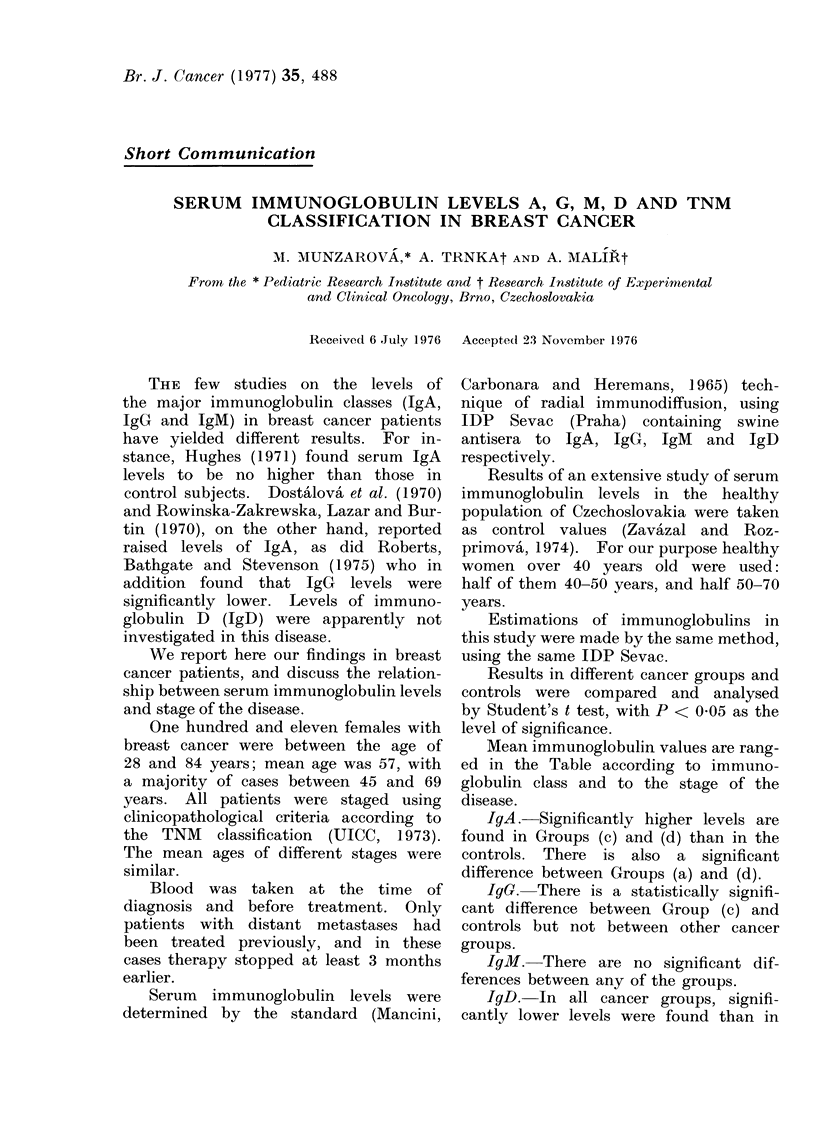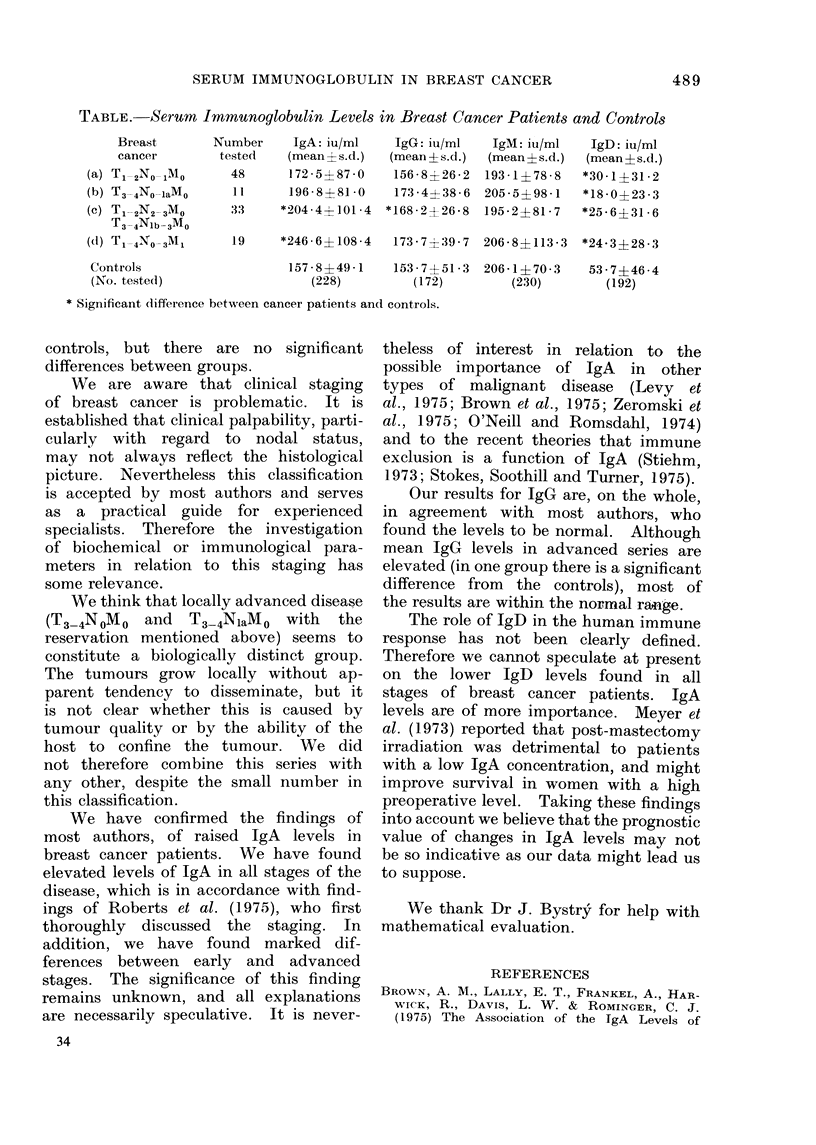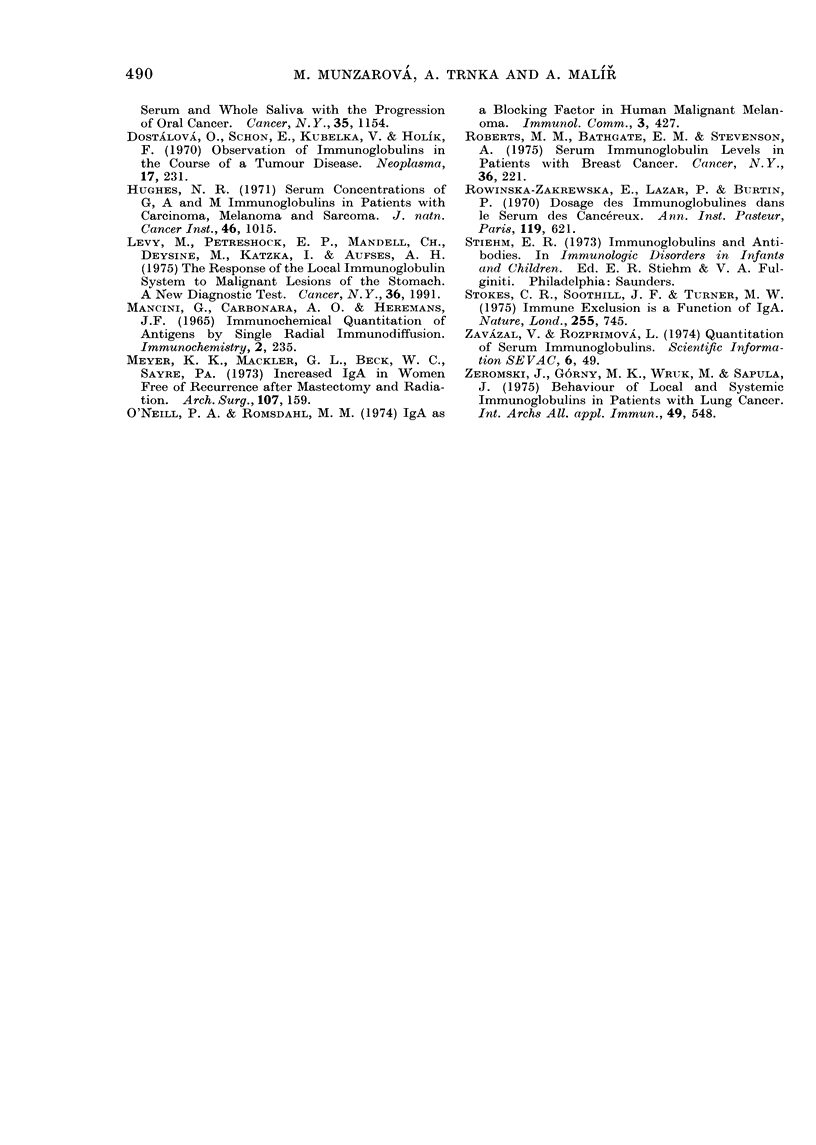# Serum immunoglobulin levels A, G, M, D and TNM classification in breast cancer.

**DOI:** 10.1038/bjc.1977.73

**Published:** 1977-04

**Authors:** M. Munzarovà, A. Trnka, A. Malìr


Br. J. Cancer (1977) 35, 488

### Short Communication

### SERUM IMMUNOGLOBULIN LEVELS A, G, M, D AND TNM CLASSIFICATION IN BREAST CANCER

M. MUNZAROVÁ,* A. TRNKA† AND A. MALÍŘ†

*From the * Pediatric Research Institute and † Research Institute of Experimental and Clinical Oncology, Brno, Czechoslovakia*

Received 6 July 1976 Accepted 23 November 1976

THE few studies on the levels of the major immunoglobulin classes (IgA, IgG and IgM) in breast cancer patients have yielded different results. For instance, Hughes (1971) found serum IgA levels to be no higher than those in control subjects. Dostálová *et al.* (1970) and Rowinska-Zakrewska, Lazar and Burtin (1970), on the other hand, reported raised levels of IgA, as did Roberts, Bathgate and Stevenson (1975) who in addition found that IgG levels were significantly lower. Levels of immunoglobulin D (IgD) were apparently not investigated in this disease.

We report here our findings in breast cancer patients, and discuss the relationship between serum immunoglobulin levels and stage of the disease.

One hundred and eleven females with breast cancer were between the age of 28 and 84 years; mean age was 57, with a majority of cases between 45 and 69 years. All patients were staged using clinicopathological criteria according to the TNM classification (UICC, 1973). The mean ages of different stages were similar.

Blood was taken at the time of diagnosis and before treatment. Only patients with distant metastases had been treated previously, and in these cases therapy stopped at least 3 months earlier.

Serum immunoglobulin levels were determined by the standard (Mancini, Carbonara and Heremans, 1965) technique of radial immunodiffusion, using IDP Sevac (Praha) containing swine antisera to IgA, IgG, IgM and IgD respectively.

Results of an extensive study of serum immunoglobulin levels in the healthy population of Czechoslovakia were taken as control values (Zavázal and Rozprimová, 1974). For our purpose healthy women over 40 years old were used: half of them 40–50 years, and half 50–70 years.

Estimations of immunoglobulins in this study were made by the same method, using the same IDP Sevac.

Results in different cancer groups and controls were compared and analysed by Student's $t$ test, with $P < 0{\cdot}05$ as the level of significance.

Mean immunoglobulin values are ranged in the Table according to immunoglobulin class and to the stage of the disease.

*IgA.*—Significantly higher levels are found in Groups (c) and (d) than in the controls. There is also a significant difference between Groups (a) and (d).

*IgG.*—There is a statistically significant difference between Group (c) and controls but not between other cancer groups.

*IgM.*—There are no significant differences between any of the groups.

*IgD.*—In all cancer groups, significantly lower levels were found than in

TABLE.—*Serum Immunoglobulin Levels in Breast Cancer Patients and Controls*

| Breast cancer | Number tested | IgA: iu/ml (mean ± s.d.) | IgG: iu/ml (mean ± s.d.) | IgM: iu/ml (mean ± s.d.) | IgD: iu/ml (mean ± s.d.) |
|---|---|---|---|---|---|
| (a) $T_{1-2}N_{0-1}M_0$ | 48 | 172·5 ± 87·0 | 156·8 ± 26·2 | 193·1 ± 78·8 | *30·1 ± 31·2 |
| (b) $T_{3-4}N_{0-1a}M_0$ | 11 | 196·8 ± 81·0 | 173·4 ± 38·6 | 205·5 ± 98·1 | *18·0 ± 23·3 |
| (c) $T_{1-2}N_{2-3}M_0$ $T_{3-4}N_{1b-3}M_0$ | 33 | *204·4 ± 101·4 | *168·2 ± 26·8 | 195·2 ± 81·7 | *25·6 ± 31·6 |
| (d) $T_{1-4}N_{0-3}M_1$ | 19 | *246·6 ± 108·4 | 173·7 ± 39·7 | 206·8 ± 113·3 | *24·3 ± 28·3 |
| Controls (No. tested) | | 157·8 ± 49·1 (228) | 153·7 ± 51·3 (172) | 206·1 ± 70·3 (230) | 53·7 ± 46·4 (192) |

* Significant difference between cancer patients and controls.

controls, but there are no significant differences between groups.

We are aware that clinical staging of breast cancer is problematic. It is established that clinical palpability, particularly with regard to nodal status, may not always reflect the histological picture. Nevertheless this classification is accepted by most authors and serves as a practical guide for experienced specialists. Therefore the investigation of biochemical or immunological parameters in relation to this staging has some relevance.

We think that locally advanced disease ($T_{3-4}N_0M_0$ and $T_{3-4}N_{1a}M_0$ with the reservation mentioned above) seems to constitute a biologically distinct group. The tumours grow locally without apparent tendency to disseminate, but it is not clear whether this is caused by tumour quality or by the ability of the host to confine the tumour. We did not therefore combine this series with any other, despite the small number in this classification.

We have confirmed the findings of most authors, of raised IgA levels in breast cancer patients. We have found elevated levels of IgA in all stages of the disease, which is in accordance with findings of Roberts *et al.* (1975), who first thoroughly discussed the staging. In addition, we have found marked differences between early and advanced stages. The significance of this finding remains unknown, and all explanations are necessarily speculative. It is nevertheless of interest in relation to the possible importance of IgA in other types of malignant disease (Levy *et al.*, 1975; Brown *et al.*, 1975; Zeromski *et al.*, 1975; O'Neill and Romsdahl, 1974) and to the recent theories that immune exclusion is a function of IgA (Stiehm, 1973; Stokes, Soothill and Turner, 1975).

Our results for IgG are, on the whole, in agreement with most authors, who found the levels to be normal. Although mean IgG levels in advanced series are elevated (in one group there is a significant difference from the controls), most of the results are within the normal range.

The role of IgD in the human immune response has not been clearly defined. Therefore we cannot speculate at present on the lower IgD levels found in all stages of breast cancer patients. IgA levels are of more importance. Meyer *et al.* (1973) reported that post-mastectomy irradiation was detrimental to patients with a low IgA concentration, and might improve survival in women with a high preoperative level. Taking these findings into account we believe that the prognostic value of changes in IgA levels may not be so indicative as our data might lead us to suppose.

We thank Dr J. Bystrý for help with mathematical evaluation.

REFERENCES

BROWN, A. M., LALLY, E. T., FRANKEL, A., HARWICK, R., DAVIS, L. W. & ROMINGER, C. J. (1975) The Association of the IgA Levels of

Serum and Whole Saliva with the Progression of Oral Cancer. *Cancer, N.Y.*, **35**, 1154.

DOSTÁLOVÁ, O., SCHON, E., KUBELKA, V. & HOLÍK, F. (1970) Observation of Immunoglobulins in the Course of a Tumour Disease. *Neoplasma*, **17**, 231.

HUGHES, N. R. (1971) Serum Concentrations of G, A and M Immunoglobulins in Patients with Carcinoma, Melanoma and Sarcoma. *J. natn. Cancer Inst.*, **46**, 1015.

LEVY, M., PETRESHOCK, E. P., MANDELL, CH., DEYSINE, M., KATZKA, I. & AUFSES, A. H. (1975) The Response of the Local Immunoglobulin System to Malignant Lesions of the Stomach. A New Diagnostic Test. *Cancer, N.Y.*, **36**, 1991.

MANCINI, G., CARBONARA, A. O. & HEREMANS, J.F. (1965) Immunochemical Quantitation of Antigens by Single Radial Immunodiffusion. *Immunochemistry*, **2**, 235.

MEYER, K. K., MACKLER, G. L., BECK, W. C., SAYRE, PA. (1973) Increased IgA in Women Free of Recurrence after Mastectomy and Radiation. *Arch. Surg.*, **107**, 159.

O'NEILL, P. A. & ROMSDAHL, M. M. (1974) IgA as a Blocking Factor in Human Malignant Melanoma. *Immunol. Comm.*, **3**, 427.

ROBERTS, M. M., BATHGATE, E. M. & STEVENSON, A. (1975) Serum Immunoglobulin Levels in Patients with Breast Cancer. *Cancer, N.Y.*, **36**, 221.

ROWINSKA-ZAKREWSKA, E., LAZAR, P. & BURTIN, P. (1970) Dosage des Immunoglobulines dans le Serum des Cancéreux. *Ann. Inst. Pasteur, Paris*, **119**, 621.

STIEHM, E. R. (1973) Immunoglobulins and Antibodies. In *Immunologic Disorders in Infants and Children.* Ed. E. R. Stiehm & V. A. Fulginiti. Philadelphia: Saunders.

STOKES, C. R., SOOTHILL, J. F. & TURNER, M. W. (1975) Immune Exclusion is a Function of IgA. *Nature, Lond.*, **255**, 745.

ZAVÁZAL, V. & ROZPRIMOVÁ, L. (1974) Quantitation of Serum Immunoglobulins. *Scientific Information SEVAC*, **6**, 49.

ZEROMSKI, J., GÓRNY, M. K., WRUK, M. & SAPULA, J. (1975) Behaviour of Local and Systemic Immunoglobulins in Patients with Lung Cancer. *Int. Archs All. appl. Immun.*, **49**, 548.